# Selenium serum levels in patients with SARS-CoV-2 infection: a systematic review and meta-analysis

**DOI:** 10.1017/jns.2023.69

**Published:** 2023-07-26

**Authors:** Nuria Renata Roldán-Bretón, Adriana Guadalupe Capuchino-Suárez, María Esther Mejía-León, Carlos Olvera-Sandoval, Dania Nimbe Lima-Sánchez

**Affiliations:** 1 Facultad de Medicina, Universidad Autónoma de Baja California, Mexicali, Baja California, Mexico; 2 Universidad Nacional Autonoma de Mexico, Ciudad de Mexico, Mexico; 3 Department of Biomedical Informatics, Universidad Nacional Autonoma de Mexico, Ciudad de Mexico, Mexico

**Keywords:** Selenium, COVID-19, Micronutrients, Clinical severity

## Abstract

The nutritional status is a determinant of the immune response that promotes a cellular homeostasis. In particular, adequate selenium levels lead to a better antioxidant and immune response. The aim of this work is to assess whether blood selenium levels, at time of SARS-CoV-2 infection, have an impact on the development and severity of COVID-19. A systematic review and meta-analysis of comparative and descriptive studies using MeSH terms, selenium and COVID-19 was performed. We searched bibliographic databases up to 17 July 2022 in PubMed and ScienceDirect. Studies that reported data on blood selenium levels were considered. A total of 629 articles were examined by abstract and title, of which 595 abstracts were read, of which 38 were included in the systematic review and 11 in the meta-analysis. Meta-analysis was conducted to mean difference (MD) with a 95 % confidence interval (CI), and heterogeneity was tested by *I*^2^ with random factors with a MD between selenium levels, mortality, morbidity and healthy subjects with a *P*-value of 0⋅05. Selenium levels were higher in healthy people compared to those in patients with COVID-19 disease (six studies, random effects MD: test for overall effect *Z* = 3⋅28 (*P* = 0⋅001), 97 % CI 28⋅36 (11⋅41–45⋅31), *P* < 0⋅00001), but without difference when compared with the degree of severity in mild, moderate or severe cases. In conclusion, the patients with active SARS-CoV-2 infection had lower selenium levels than the healthy population. More studies are needed to evaluate its impact on clinical severity through randomised clinical trials.

## Introduction

Coronavirus disease 2019 (COVID-19), caused by the severe acute respiratory syndrome coronavirus 2 (SARS-CoV-2), has been the subject of several study approaches to avoid or minimise the severity of the disease, considering that this condition has potentially severe effects at the systemic level, damaging organs such as lungs, heart, esophagus, kidneys, bladder and ileum^(1,2)^.

Selenium is an essential micronutrient with a normal concentration ranging from 3 to 20 mg in a human organism, and a distribution of 46⋅9 % in skeletal muscle, 4 % in the kidney and the rest is ubiquitous^(3)^. The maximum activity of glutathione peroxidase, an enzyme with antioxidant effect, is achieved with a daily intake of 55–70 μg (0⋅70–0⋅89 μmol) of selenium and is associated with plasma selenium in the normal physiological range of 90–125 μg/l^(4)^. Selenium can cause toxicity when ingested above 800 μg/d, with the safe upper limit defined at 400 μg/d^(5)^. This trace element appears to play an important role in protecting the respiratory system, particularly against viral infections^(6,7)^. Beck *et al.*^(8)^ found that selenium deficiency significantly increased susceptibility to influenza virus-induced lung pathology, in association with overexpression of proinflammatory cytokines. These findings are consistent with the observation of lower levels of interferon-γ (IFN-γ) and tumour necrosis factor (TNF-α), as well as a reduced survival rate in selenium-deficient mice infected with influenza virus, when compared to mice that were supplemented with sodium selenite. In turn, selenium treatment was shown to increase the expression of interferon genes (IFN-α, IFN-β and IFN-γ) in response to avian influenza virus (H9N2)^(9,10)^. This interaction prompts us to seek comparative studies that measure selenium levels and the morbidity and mortality of COVID-19 in humans. The aim of this work is to assess whether blood selenium levels, at time of SARS-CoV-2 infection have an impact on the development and severity of SARS-CoV-2 infection.

## Methods

A systematic review and meta-analysis about studies of blood selenium levels and COVID-19, having as inclusion criteria scientific articles published from January 2020 to July 2022, following the Cochrane methodology with the following PICO question.

P: Patients with COVID-19

I: Selenium deficiency

C: Healthy individuals

O: Risk factor in clinical severity

Studies that reported the role of serum selenium level in COVID-19 infection and progression were included. We searched papers in PubMed and ScienceDirect databases using MeSH keywords/terms, such as: (‘selenium’[MeSH Terms] OR ‘selenium’[All Fields] OR ‘selenium s’[All Fields] OR ‘seleniums’[All Fields]) AND (‘sars cov 2’[MeSH Terms] OR ‘sars cov 2’[All Fields] OR ‘covid’[All Fields] OR ‘covid 19’[MeSH Terms] OR ‘covid 19’[All Fields]). Searches were not restricted by language, study design or country of origin.

**Fig. 1.**
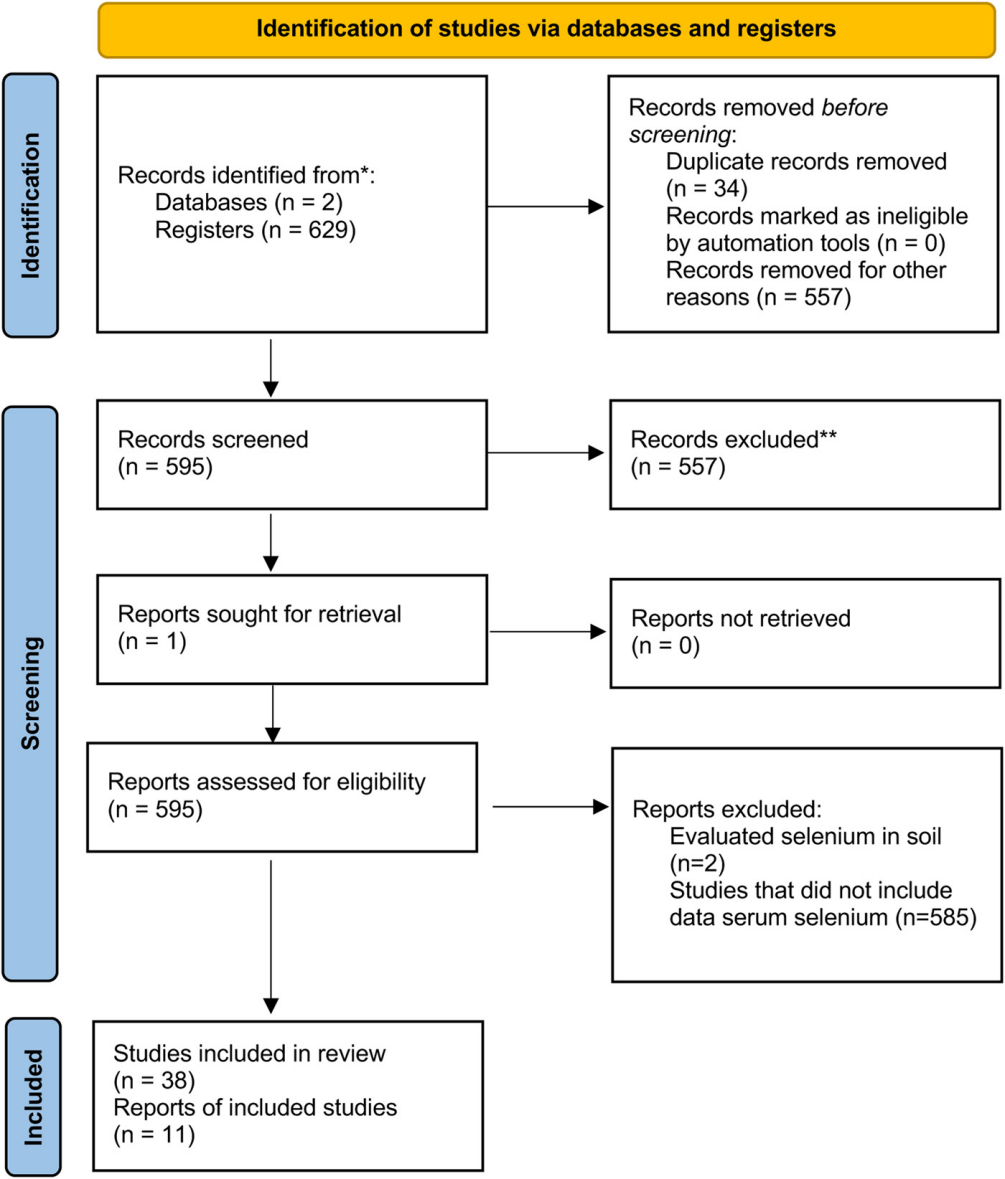
Study selection flowchart in the PRISMA diagram.

Two reviewers independently performed title-abstract screening on all selected studies, then the full-text of the selected articles were reviewed. In cases of duplicate information, only one paper was considered. Each reviewer analysed the data about serum selenium levels in confirmed COVID-19 subjects. The primary outcome of interest was mortality. The secondary outcomes were data on serum selenium levels in healthy individuals compared to those in COVID-19 patients during the onset and progression of the disease (e.g. mild, moderate and severe). Data from included studies were separately extracted, considering key characteristics such as author, publication year, country, type of study, sample size, laboratory findings on selenium levels and final clinical outcomes. The exclusion criteria were articles published after the evaluated period or that did not meet the described search criteria. Narrative reviews that cited the comparative articles already referenced or those that did not report data on the outcomes evaluated were also excluded as were those studies conducted *in vitro* or on minor species. After performing the database search and reading the abstracts of the articles that met the inclusion criteria, we applied the elimination criteria specified in the PRISMA diagram (Fig. 1) and removed any duplicate articles. Two reviewers performed full-text reading of the remaining articles and made a checklist to reach a consensus on those papers that met the selection criteria. Subsequently, we used the Newcastle-Ottawa Scale (NOS) system to estimate the quality of the included studies. The NOS system was defined with three components: (1) selection of study groups, (2) comparability of groups and (3) ascertainment of outcomes. Added scores ranged from 0 to 9 points (from the lowest to the highest). Any disagreements about the methodological quality of the results were resolved by discussion. Meta-analysis was conducted to mean difference (MD) with 95 % confidence interval (CI), and heterogeneity was tested by *I*^2^. To ensure compliance with Cochrane guidelines, we used RevMan 5.1 to perform a meta-analysis process. A summary table of the findings was reported with the data obtained (Table 1). For those articles that did not report the mean values and standard deviation of serum selenium levels, we electronically contacted the corresponding author but only one author reported the data to us and it was included in the study^(17)^.

**Table 1.** Description of eligible studies on the effects of selenium against COVID-19 included in the meta-analysis

Author, year	Study design	Participants/country	Selenium normal range	Outcomes
Majeed, 2021^(11)^	Cross-sectional study	30 COVID-19 patients from India	The selenium levels of thirty healthy subjects were the reference range	The COVID-19 patients showed significantly lower selenium levels of 69⋅2 ± 8⋅7 ng/ml than healthy subjects 79⋅1 ± 10⋅9 ng/ml (*P* < 0⋅0003)
Younesian, 2022^(12)^	Cross-sectional study	50 COVID-19 patients in Iran hospital	The selenium levels of thirty healthy subjects were the reference range	The selenium level was lower in COVID-19 patients (77⋅8 μg/l ± 13⋅9 μg/l) as compared to healthy control individuals (91⋅7 μg/l ± 16⋅7 μg/l) (*P* < 0⋅05), but there was no significant difference between the survivor and non-survivor groups
Skalny, 2021^(13)^	Prospective observational study	150 COVID-19 patients in a clinical centre of Russia	The selenium levels of forty-four healthy subjects were the reference range	43⋅4 % of COVID-19 patients with low values (<2⋅5 of the reference population) were associated with higher mortality
Moghhaddam, 2020^(14)^	Observational, cross-sectional study	33 COVID-19 patients of a hospital in German	Classifies a normal selenium status when residing in the reference range of 45⋅7–131⋅6 μg/l for serum selenium in European people	64⋅7 % of the samples of deceased COVID-19 patients showed selenium deficiency, whereas 39⋅3 % of the samples from the survivors were classified as selenium-deficient
Kocak, 2021^(15)^	Cross-sectional study	60 COVID-19 patients in a research hospital of Turkey	The selenium levels of thirty-two healthy subjects were the reference range	Selenium level was lower than the comparison group, 215⋅34 ± 49⋅83 ng/ml *v.* 255⋅23 ± 42⋅67 ng/ml, *P* < 0⋅001, COVID-19 positive patients and healthy subjects, respectively
Chanihoon, 2022^(16)^	Comparative	63 patients with COVID-19 and 87 referents group in Pakistan	Ranges were compared between COVID-19 patients and healthy patients without considering a reference range	Selenium serum level in COVID-19 patients non-smokers 53⋅0 ± 6⋅09 μg/l and referents group non-smokers 105 ± 10⋅4 μg/l. Low concentrations of essential selenium from healthy smokers and non-smokers, might contribute to the recurrence of COVID-19
Alkattan, 2021^(17)^	Cross-sectional study	80 COVID-19 patients in a Saudi Arabian hospital	Normal range of selenium in blood serum 70–150 μg/l were considering	Studies groups were divided into non-severe cases and severe cases. the mean selenium in both study groups was within the normal range (138 μg/l). In selenium mean serum level between non-severe and severe cases, which showed a significant elevation of selenium level among severe cases of COVID-19 patients (134 *v.* 162 μg/l, *P*-value < 0⋅0001)
Al-Saleh, 2022^(18)^	Cross-sectional study	155 COVID-19 patients in a Saudi Arabian hospital	Normal range of selenium in blood serum 70⋅08 μg/–119⋅69 μg/l were considering	The serum selenium level was evaluated in four classes of groups: asymptomatic (86⋅56 ± 18⋅9 5 μg/l), mild (78⋅36 ± 18⋅04 μg/l), moderate (87⋅51 ± 19⋅26 μg/l) and severe (76⋅6 ± 23⋅54 μg/l). The investigators used separate linear regression models. Parameters showed a decrease in selenium (*β* = −0⋅203, 95 % CI,0⋅308, −0⋅015, *P* = 0⋅074) in severe COVID-19 patients compared to asymptomatic
Baguer, 2021^(19)^	Single-centered prospective, observational study cohort	114 patients admitted to severe intensive care units (ICUs) and corresponding 112 sex and aged-matched non-ICU ward patients	Normal range of selenium serum 70–150 μg/l were considering	The serum values for selenium in both groups were reported as 130⋅19 ± 3⋅19 μg/l ICUs patients compared with non-ICU patients (123⋅06 ± 2⋅58 μg/l, *P* = 0⋅084)
Razeghi, 2021^(20)^	Observational prospective study	84 COVID-19 patients in Sina Hospital	Normal range of selenium serum 70–150 μg/l were considering	Significant negative association between serum selenium level and COVID-19 severity (standardised coefficient = − 0⋅28, *P*-value = 0⋅01). The association did not remain significant after adjusting for potential confounding factors
Voelkle, 2022^(21)^	Observational Cohort Study	57 COVID-19 patients from Cantonal Hospital Aarau, Switzerland	Normal range of selenium serum < 0⋅94 μmol/l were considering	Selenium deficiencies were most prevalent, 51 %. Selenium levels were lower when patients had more micronutrient deficiencies (*P* < 0⋅01). In patients with a single micronutrient deficiency, selenium deficiency was most prevalent (*n* = 5, 50 %)

## Results

The initial search yielded 629 articles, with duplicate studies removed, resulting in 595 remaining. Following the inclusion–exclusion criteria, 38 studies were selected for systematic review. This included 808 confirmed cases of COVID-19 and comprised of 11 studies that reported the mean and standard deviation data serum selenium level (Fig. 1).

Of the thirty-eight studies, those that did not satisfy the criteria for meta-analysis were included in the systematic review.

1. Studies that evaluated the role of serum selenium levels in COVID-19 mortality and lethality.

One of the studies that evaluated the role of selenium in the progression, of the disease considered the presence of selenium in the soil. It was conducted in China where the fatality rates of COVID-19 were compared with the status of selenium in soil of its territory by county. Selenium measurement was classified into three categories: areas without selenium deficiency (>0⋅06 ppm), areas with moderate selenium deficiency (0⋅03–0⋅06 ppm) and areas with severe selenium deficiency (<0⋅03 ppm). They found that selenium-deficient regions had a 3⋅16 % case fatality rate *v*. 1⋅17 % in other areas (*P* = 0⋅002), with an incidence rate of 3⋅88 (95 % CI 1⋅21, 12⋅52). For this study, possibly related socio-demographic co-variables were controlled, such as age, gross domestic product per capita, population density, medical access, number of beds and number of medical staffs. This is largely consistent with the classification based on topsoil selenium content (Spearman's correlation coefficient of 0⋅46, *P* < 0⋅001). Among the top ten cities with the highest fatality rates (3⋅70–8⋅51 %), four cities were grouped as severe soil selenium-deficient regions and five as moderate soil selenium-deficient regions. These results suggest that regional selenium deficiency could be associated with a higher lethality of COVID-19 infection^(22)^.

Also, in China, a retrospective analysis was carried out in which the cure rate of patients who had COVID-19 was correlated with selenium levels measured in hair. A significant association between cure rate and background selenium status in cities outside Hubei were detected (*R*^2^ = 0⋅72, *F* test, *P* < 0⋅0001). In this study, provinces or municipalities with more than 200 cases and cities with more than 40 cases were included. The researchers concluded that high selenium levels are associated with a higher cure rate. There are biases regarding the selection of data, so this evidence should be taken with reserve^(23)^.

However, in addition to soil and hair, other studies began to analyse the presence of selenium and its biomarkers such as selenoprotein P (SELENOP) in blood, evaluating its role in COVID-19.

Another study in Germany was conducted to evaluate different micronutrient concentrations and compare them between patients who had survived COVID-19 and those who had not. The authors found that the combination of copper deficiency and SELENOP are parameters that can contribute to predicting disease survival^(24)^. These findings are replicated in the study by Heller *et al.*^(25)^, which shows that patients with COVID-19 had low selenium levels and higher rate mortality. In other study conducted in Iran, blood selenium levels were evaluated in patients suspected of having COVID-19. There also evaluated other variables such as white blood cell count, lymphocytes, neutrophils, platelets, lactate dehydrogenase, length of hospitalisation and age in order to understand the inflammatory status of the disease. In this study, a control group of fifty individuals, who were classified by age and gender, was considered. Of the group of COVID-19 patients, thirteen died and thirty-seven recovered. The median recovery time was 6 d, and the median time to death was 12 d. The average age of the patients was 56 years and most of them were men. Serum selenium levels were significantly different in patients with COVID-19 *v.* control group (77⋅8 ± 13⋅9 μg/l *v.* 91⋅7 ± 16⋅7 μg/l) but it should be noted that there were no significant differences between survivors and non-survivors (77⋅9 ± 14⋅2 *v.* 77⋅2 ± 12⋅3 μg/l). This study shows that selenium levels were lower in COVID-19 patients *v*. the healthy individuals^(26)^. Other group of investigation, measured blood selenium in 226 patients with COVID-19, confirmed with PCR and that were hospitalised at the moment of the study. Patients were classified into severe and not severe according to the type of hospitalisation, inside and outside the intensive care unit (ICU). When considering the normal reference values of selenium in blood from 70 to 150 μg/l, no significative differences were detected when comparing patients in ICU (130⋅19 ± 3⋅19 μg/l) and non-ICU (123⋅06 ± 2⋅58 μg/l), as well as patients who died (129⋅15 ± 3⋅91 μg/l) *v*. those who recovered (125⋅77 ± 2⋅41 μg/l) 56 (24⋅78 %)^(19)^. In this study, the selenium levels in COVID-19 patients and healthy individuals were not compared. Finally, in an observational study of 169 COVID-19 participants, Maares *et al.*^(27)^ reported positive associations between serum selenium and serum SELENOP levels with free zinc concentrations, compared between survivors and non-survivors, reporting higher concentrations of both biomarkers in those patients who survived the disease.

1. Studies that evaluated the role of serum selenium levels in COVID-19 morbidity.

In 2020, a study was carried out in South Korea with a sample of fifty COVID-19 patients (twenty-nine men and twenty-one women) from whom blood was drawn in the first stage of infection and concentrations of different vitamins and selenium were quantified. Of the obtained results, the most outstanding were in relation to vitamin D and selenium, since a vitamin D deficiency was reported in both COVID-19 patients and participants from the control group. In relation to selenium, it was found that 42 % of patients with COVID-19 had selenium levels below the limit (95 ng/ml), with an average of 98⋅3 ng/ml (range 90⋅3–107⋅6 ng/ml). From this percentage, eight patients were without any complications and thirteen were with pneumonia some of them required oxygen supplementation, high-flow nasal cannula, mechanical ventilator and extracorporeal membrane oxygenations. Of the pneumonia patients, four died from complications and all of them had selenium deficiency. The researchers showed with their study a general trend of deficiency and the importance of nutrition in the prevention of severe cases of COVID-19^(28)^. These findings were replicated in a comparative study that evaluated eighty patients who had previously suffered COVID-19 and forty patients who coursed with acute COVID-19 at the moment of the study. The levels of selenium were low in both groups, with a greater severity in the acute phase of COVID-19 (*n* 40, 69⋅7 ± 20⋅8 μg/l), than in the recovered ones (Spring wave *n* 40, 84⋅6 μg/l, sd = 20⋅7 and Autumn wave *n* 40, 88⋅2 μg/l, sd = 27⋅2)^(29)^. Similarly, a study conducted in COVID-19 patients between January and May 2020 showed that patients with severe COVID-19 were found to be decreased compared to patients suffering from mild COVID-19 (*P* = 0⋅0024)^(30)^.

Razeghi *et al.*^(20)^ found a negative association between the levels of selenium and zinc with respect to the degree of clinical severity of COVID-19 in eighty-four patients. In this study, patients with mild disease (*n* 38) presented serum selenium concentrations of 47⋅07 ± 20⋅82 ng/ml, while those with moderate (*n* 27) and severe disease (*n* 19) had levels of 47⋅36 ± 25⋅6 ng/ml and 29⋅86 ± 11⋅48 ng/ml, respectively. It is important to highlight that in all cases lower than normal values reported by the authors (70–150 ng/ml) were found.

1. Studies that compared selenium levels in healthy and sick individuals with COVID-19.

A study conducted in southern India found low selenium levels in patients with COVID-19. The analysis was performed by plasma mass spectrometry of healthy individuals (control group) and COVID-19 patients with mild symptoms without hypoxia, excluding asymptomatic patients or patients who received nutrient supplementation at the moment of the study. The average selenium level in COVID-19 patients was 69⋅2 ± 8⋅7 ng/ml while in the control group was 79⋅1 ± 10⋅9 ng/ml. In this study, other variables such as age, sex and body mass index were assessed, which allowed to rule out a correlation of them with selenium status. It is noteworthy that men showed a greater trend of low selenium levels when compared to women in the study (79⋅4 ± 9⋅2 *v.* 68⋅4 ± 8⋅2 ng/ml, *P* < 0⋅01)^(11)^.

Similarly, a study was conducted in Turkey with sixty cases of COVID-19, mostly men with a mean age of 48⋅8 years (*n* 32) and twenty-two healthy individuals, the majority being women with a mean age of 45⋅5 years (*n* 21) as a control group. Most of the patients developed moderate symptoms (*n* 28). Mean selenium levels in patients were 215⋅34 ± 49⋅83 ng/ml and in the controls, they were 255⋅23 ± 42⋅67 ng/ml, *P* > 0⋅001. The authors suggest that this difference could explain, in part, the disease process in COVID-19 patients^(15)^.

Another research group classified the 150 COVID-19 patients in their study by degree of severity, into mild, moderate and severe, paired with 44 healthy subjects. With this design, they identified that selenium levels in the control group were 102 ± 16 μg/l and that these values were decreasing in relation to the severity in patients with COVID-19. Thus, in mild cases, the reported levels were 93 ± 20 μg/l, in moderate cases 90 ± 22 μg/l and in severe cases 67 ± 31 μg/l, the difference between severe cases and the control group was significant (*P*  = <0⋅001) and was the difference between patients with moderate symptoms and the control group (*P* = 0⋅047). An inverse correlation was found between lung damage and selenium levels (*r* = 0⋅297, *P* = 0⋅074) as well as a positive correlation between selenium and oxygen saturation levels (*r* = 0⋅195, *P* = 0⋅007). In the regression model, they found that selenium levels were a significant negative predictor of lung damage^(13)^.

In a small study, where they evaluated fifteen patients with COVID-19 who underwent medical treatment with Favipravir 200 mg tablet (2 × 1600 mg for loading dose, 2 × 600 mg for maintenance dose up to 5–10 d) and Hydroxychloroquine 200 mg tablet (2 × 400 mg for loading dose and 2 × 200 mg for maintenance dose up to 5–10 d) were evaluated. Selenium levels were initially low in most of the patients, and improved slightly during treatment (86⋅7–73⋅3 %). The authors state that the use of antivirals can improve inflammation and be the pathway by which selenium levels increase, but more studies are needed to confirm this^(31)^.

1. Other findings

In an ecological study by the European Food Safety Authority, the role of six vitamins (D, A, C, Folate, B6 and B12) and four minerals (zinc, iron, copper and selenium) in the function of the immune system, in order to determine its current importance in relation to the COVID-19 pandemic. In this study, nutritional status was correlated with epidemiological data on COVID-19 in European countries, specifically a negative correlation between selenium and COVID-19 incidence and deaths. In addition to this, the relationship of some genetic variants and how they influence the nutritional status of these nutrients was included. The importance of vitamin D and iron in overcoming COVID-19 is highlighted, but no data is shown indicating specific recommended intake for this situation^(32)^.

The study by Notz *et al.* sought to evaluate the impact of selenium supplementation in patients with severe COVID-19, hospitalised in the ICU. In these patients, a dose of 1 mg of selenium daily was administered intravenously along with parenteral nutrition. Selenium and SELENOP levels were evaluated both on admission and on discharge from the hospital. Twenty-two patients (women, *n* 8; men, *n* 14) with a mean age of 60⋅5 years were included in the sample. Of these patients, 64 % (14) survived and seven died; furthermore, upon admission to intensive care, eight patients had a selenium deficiency and SELENOP. With daily supplementation, eleven patients normalised their levels in a range of 10–14 d. They also identified that selenium levels were inversely correlated with C-reactive protein, procalcitonin, and had a positive association with NK cells number^(33)^.

On the other hand, Erol *et al.* evaluated pregnant COVID-19 patients according to their trimesters of gestation in relation to pregnant women without COVID-19 as a control group. In the first trimester, twenty-four women with COVID-19 were evaluated compared to twenty-six in the control group, in the second trimester, there were twenty-six patients and twenty-two controls and in third trimester, with twenty-one patients with COVID-19 and twenty-two controls participated. Selenium levels were significantly reduced in the second (36⋅03 ± 9⋅68 *v.* 46⋅15 ± 8⋅15 maternal selenium level, mcg/l, *P* = 0⋅001) and third trimesters (27⋅01 ± 7⋅82 *v.* 36⋅15 ± 6⋅25 maternal selenium level, mcg/l, *P* < 0⋅01). However, there were no differences in the first trimester (46⋅52 ± 8⋅17 *v.* 44⋅59 ± 8⋅4 maternal selenium level, mcg/l). Serum selenium levels in women with COVID-19 decreased as the weeks of gestation progressed (*r* = –0⋅541, *P* < 0⋅001)^(34)^.

In a cohort study where the immune response in healthcare personnel who received two doses of the BNT162b2 mRNA vaccine were evaluated, there was no significant association of Se status with the humoral immune response to the vaccine^(35)^.

After screening and selection, articles were classified and ordered according to their study design, population, normal selenium range and outcomes, as shown in Table 1.

### Meta-analysis

A meta-analysis was performed using a random effects model and two comparisons: (1) reported selenium levels in blood of healthy subjects and patients with COVID-19 and (2) selenium levels in blood compared at different stages of disease severity, according to presented symptoms. Illness severity was classified into four groups based on the Adult Guidelines for the Management of Coronavirus Disease 2019 for Classification: Asymptomatic Patients with no signs or symptoms of infection; Mild. Patients with upper respiratory tract infection symptoms and other mild symptoms (including fever and gastrointestinal symptoms) without evidence of pneumonia; Moderate. Patients with hypoxia with oxygen saturation less than 93 % at rest or presence of pneumonia not requiring ICU admission; Severe. Patients with pneumonia requiring ICU admission or any of the following: Critical respiratory failure requiring mechanical ventilation, septic shock or multiorgan dysfunction^(17)^.

**Fig. 2.**
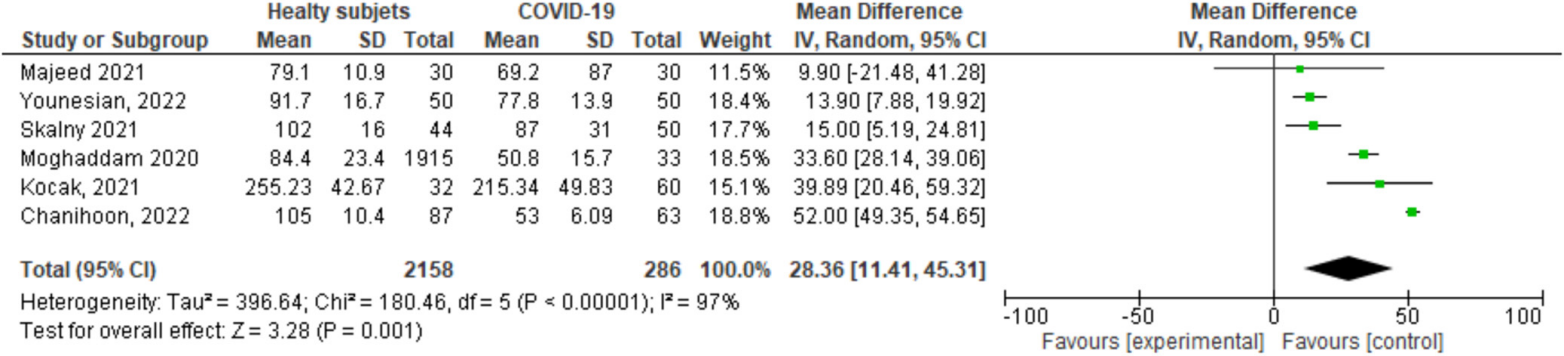
Selenium serum levels in COVID-19 patients and healthy individuals.

A random effects analysis was performed to evaluate the difference in means, considering the Heterogeneity Index. This analysis resulted in a significant effect size of 3⋅28 (*P* = 0⋅001), which showed that selenium levels in healthy individuals were significantly higher than those in COVID-19 patients. The results obtained from each study are consistent although heterogeneous (*I*^2^ = 97 %, *P* < 0⋅00001) (Fig. 2).

**Fig. 3.**
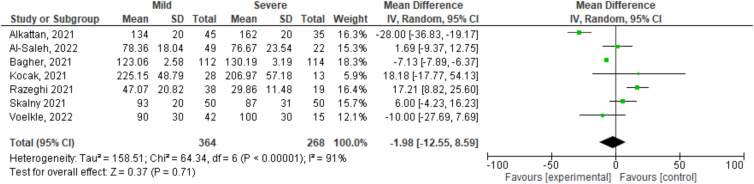
Degrees of clinical severity of COVID-19 and selenium serum levels reported in the included studies.

The heterogeneity of the data among the group of patients with moderate disease was high, since selenium levels were lower in this group compared to the selenium levels of asymptomatic patients. Therefore, the values of the meta-analysis of the comparison of selenium levels in the different stages of the disease were not significant (test for overall effect *Z* = 0⋅37, *P* = 0⋅71; 95 % CI –1⋅98 (−12⋅55, 8⋅59) and a heterogeneity *I*^2^ = 91 %, *P* < 0⋅00001) (Fig. 3).

## Discussion

Our findings suggest that selenium levels are lower in SARS-CoV-2 infected patients compared to uninfected subjects. The relationship between selenium and COVID-19 has been discussed in the several published studies^(14,23,27,36)^. The hypothesis arises from mechanistic evidence from biochemical and *in vitro* studies of SARS-CoV-2 or other respiratory viruses, showing that selenium may support the immune system function or antioxidant effect^(8,10,37,38)^. In addition, some authors reported blood selenium values in patients with different degrees of clinical severity of infection, noting that in severe disease, selenium values are very low compared to moderate and, in turn, compared to mild disease^(11,14,26,39)^. However, one study reported higher selenium values when considering all levels of severity together, although proportionally lower selenium levels were observed in patients with severe disease^(15)^.

Our research coincides with the systematic review by Fakhrolmobasheri *et al.*^(40)^, where eleven studies that evaluated selenium levels in COVID-19 patients were included; most of them cross-sectional, with selenium measured in serum. Overall, they found that selenium levels were lower in patients with COVID-19 compared to healthy individuals, but they did not perform a meta-analysis to assess the quantitative effect. Until July 2022, no other systematic reviews with meta-analysis evaluating selenium levels in COVID-19 were found. Another interesting result of Fakhrolmobasheri *et al.*^(40)^ was that showed a study that evaluated urinary selenium levels and compare them between severe and no severe COVID-19 patients (20⋅27 (13⋅53–35⋅34 μg/l *v.* 25⋅5 (19⋅04–37⋅64 μg/l), *P* = 0⋅024) but specially in fatal COVID-19 patients, there were higher urinary selenium levels compared to recovered COVID-19 patients^(41)^. We agree with the authors that these results could be caused by dysfunction in stress-related pathways or, it could be the result of drug interactions to some drugs commonly used in the treatment of patients with COVID-19, such as corticosteroids, which can interfere with selenium absorption and increase its excretion. Another hypothesis is that in patients with severe COVID-19 there is a higher probability of impaired renal function, which coincides with the higher excretion of selenium when adjusted for creatinine. This finding could explain, in part, the elevated urinary selenium in contrast to the low blood levels in these patients.

In addition to the above, we hypothesise that a COVID-19 patient with an active oxidative stress process increases the demand for selenium due to the heightened immune response that occurs in the disease. This process could lead to a reduction in low blood selenium levels. Selenium is an essential micronutrient that plays an important role in the regulation of immune function, and it is known that low selenium levels can negatively affect the immune response. Therefore, if patients with COVID-19 have an overactive immune response, a depletion of selenium levels in the body may occur and contribute to the lower levels observed in the blood. However, further research is needed to confirm this hypothesis and to determine whether other factors may also be contributing to the low selenium levels in patients with COVID-19.

The main limitation of our study is that only comparative studies were considered in the analysis since they are the ones that were published to date. Future blinded randomised clinical trials are needed to assess the effect of selenium levels on SARS-CoV-2 infection, as well as to evaluate the response to new viral variants and immunisation schemes. Another limitation of this research is that to date there were few studies that have associated SARS-CoV-2 infection with selenium levels in the blood. COVID-19 continues to be a new disease, where new related studies are published on a daily and this review only assesses works published up to July 2022. In addition, each study has factors that are not considered in their final conclusions, such as inflammatory response markers, drug treatment, virus variants, which also limit the conclusions of the study. Finally, the investigation of randomised clinical studies that can strengthen it is the levels of evidence, in different populations and with a large sample size, is considered necessary to probe the possible effect of selenium on the severity and mortality of COVID-19.

## Perspectives

So far, the pharmacological treatment for COVID-19 is still under development and research, which highlights the importance of maintaining an optimal nutritional status that allows preserving homeostasis conditions at the cellular level and thus, the individual can carry out the necessary mechanisms to combat the disease, especially with the new variants. In this review, it has been shown, with the results of the meta-analysis, that selenium levels could be the difference between acquiring the disease or not. Modifying individual vulnerability when exposed to the virus. Therefore, to maintain optimal health, it is necessary to integrate foods rich in micronutrients such as selenium into the diet to maintain nutritional balance and possibly help prevent infections such as SARS-CoV-2. However, COVID-19 is a multifactorial disease, once acquired, any risk factor can contribute to the severity of the infection, so selenium levels will not be an exclusive factor that determines the recovery capacity from the disease. Thus, it is necessary to continue research on the role of selenium to clarify its relationship with the disease in detail.

## Conclusions

Our review and meta-analysis provide compelling evidence that serum selenium is lower in COVID-19 patients compared to healthy subjects. In addition, we did not find that selenium levels will be found to be related to the different stages of the disease. However, once COVID-19 is established, changes in selenium levels at different degrees of disease severity are not consistent. Therefore, it would be interesting to assess, in future studies, the nutritional status and selenium status in COVID-19 patients to generate strong evidence that support selenium supplementation together with drug treatment, considering that selenium benefits human health in optimal amounts.

## References

[1] Hu B, Guo H, Zhou P, et al. (2020) Characteristics of SARS-CoV-2 and COVID-19. Nat Rev Microbiol 20, 141–154. 10.1038/s41579-020-00459-7 PMC7537588 33024307

[2] Devaux CA, Rolain J-M & Raoult D (2020) ACE2 receptor polymorphism: susceptibility to SARS-CoV-2, hypertension, multi-organ failure, and COVID-19 disease outcome. J Microbiol Immunol Infect 53, 425–435. 32414646 10.1016/j.jmii.2020.04.015 PMC7201239

[3] Kieliszek M (2019) Selenium – fascinating microelement, properties and sources in food. Molecules 24, 1298. 30987088 10.3390/molecules24071298 PMC6480557

[4] Kieliszek M & Błażejak S (2013) Selenium: significance, and outlook for supplementation. Nutrition 29, 713–718. 23422539 10.1016/j.nut.2012.11.012

[5] Zhang J, Saad R, Taylor EW, et al. (2020) Selenium and selenoproteins in viral infection with potential relevance to COVID-19. Redox Biol 37, 101715. 32992282 10.1016/j.redox.2020.101715 PMC7481318

[6] Avery JC & Hoffmann PR (2018) Selenium, selenoproteins, and immunity. Nutrients 10, 1203. 30200430 10.3390/nu10091203 PMC6163284

[7] Maggini S, Pierre A & Calder PC (2018) Immune function and micronutrient requirements change over the life course. Nutrients 10, 1531. 30336639 10.3390/nu10101531 PMC6212925

[8] Beck MA, Nelson HK, Shi Q, et al. (2001) Selenium deficiency increases the pathology of an influenza virus infection. FASEB J 15, 1481–1483. 11387264

[9] Shojadoost B, Kulkarni RR, Yitbarek A, et al. (2019) Dietary selenium supplementation enhances antiviral immunity in chickens challenged with low pathogenic avian influenza virus subtype H9N2. Vet Immunol Immunopathol 207, 62–68. 30593352 10.1016/j.vetimm.2018.12.002

[10] Yu L, Sun L, Nan Y, et al. (2011) Protection from H1N1 influenza virus infections in mice by supplementation with selenium: a comparison with selenium-deficient mice. Biol Trace Elem Res 141, 254–261. 20490710 10.1007/s12011-010-8726-x

[11] Majeed M, Nagabhushanam K, Gowda S, et al. (2021) An exploratory study of selenium status in healthy individuals and in patients with COVID-19 in a south Indian population: the case for adequate selenium status. Nutrition 82, 111053. 33321395 10.1016/j.nut.2020.111053 PMC7657009

[12] Younesian O, Khodabakhshi B, Abdolahi N, et al. (2022) Decreased serum selenium levels of COVID-19 patients in comparison with healthy individuals. Biol Trace Elem Res 200, 1562–1567. 34195940 10.1007/s12011-021-02797-w PMC8245273

[13] Skalny AV, Timashev PS, Aschner M, et al. (2021) Serum zinc, copper, and other biometals are associated with COVID-19 severity markers. Metabolites 11, 244. 33920813 10.3390/metabo11040244 PMC8071197

[14] Moghaddam A, Heller RA, Sun Q, et al. (2020) Selenium deficiency is associated with mortality risk from COVID-19. Nutrients 12, E2098. 10.3390/nu12072098 PMC7400921 32708526

[15] Kocak OF, Ozgeris FB, Parlak E, et al. (2021) Evaluation of serum trace element levels and biochemical parameters of COVID-19 patients according to disease severity. Biol Trace Elem Res 200, 3138–3146. 34608570 10.1007/s12011-021-02946-1 PMC8489790

[16] Chanihoon GQ, Afridi HI, Unar A, et al. (2022) Selenium and mercury concentrations in biological samples from patients with COVID-19. J Trace Elem Med Biol 73, 127038. 35863260 10.1016/j.jtemb.2022.127038 PMC9288246

[17] Alkattan A, Alabdulkareem K, Kamel A, et al. (2021) Correlation between Micronutrient plasma concentration and disease severity in COVID-19 patients. Alex J Med 57, 21–27.

[18] Al-Saleh I, Alrushud N, Alnuwaysir H, et al. (2022) Essential metals, vitamins and antioxidant enzyme activities in COVID-19 patients and their potential associations with the disease severity. Biometals 35, 125–145. 34993712 10.1007/s10534-021-00355-4 PMC8736309

[19] Bagher Pour O, Yahyavi Y, Karimi A, et al. (2021) Serum trace elements levels and clinical outcomes among Iranian COVID-19 patients. Int J Infect Dis 111, 164–168. 34454118 10.1016/j.ijid.2021.08.053 PMC8384760

[20] Razeghi Jahromi S, Moradi Tabriz H, Togha M, et al. (2021) The correlation between serum selenium, zinc, and COVID-19 severity: an observational study. BMC Infect Dis 21, 899. 34479494 10.1186/s12879-021-06617-3 PMC8414458

[21] Voelkle M, Gregoriano C, Neyer P, et al. (2022) Prevalence of micronutrient deficiencies in patients hospitalized with COVID-19: an observational cohort study. Nutrients 14, 1862. 35565831 10.3390/nu14091862 PMC9101904

[22] Zhang H-Y, Zhang A-R, Lu Q-B, et al. (2021) Association between fatality rate of COVID-19 and selenium deficiency in China. BMC Infect Dis 21, 452. 34011281 10.1186/s12879-021-06167-8 PMC8132024

[23] Zhang J, Taylor EW, Bennett K, et al. (2020) Association between regional selenium status and reported outcome of COVID-19 cases in China. Am J Clin Nutr 111, 1297–1299. 32342979 10.1093/ajcn/nqaa095 PMC7197590

[24] Hackler J, Heller RA, Sun Q, et al. (2021) Relation of serum copper status to survival in COVID-19. Nutrients 13, 1898. 34072977 10.3390/nu13061898 PMC8229409

[25] Heller RA, Sun Q, Hackler J, et al. (2021) Prediction of survival odds in COVID-19 by zinc, age and selenoprotein P as composite biomarker. Redox Biol 38, 101764. 33126054 10.1016/j.redox.2020.101764 PMC7574778

[26] Younesian O, Khodabakhshi B, Abdolahi N, et al. (2021) Decreased serum selenium levels of COVID-19 patients in comparison with healthy individuals. Biol Trace Elem Res 200, 1526–1567. 10.1007/s12011-021-02797-w PMC8245273 34195940

[27] Maares M, Hackler J, Haupt A, et al. (2022) Free zinc as a predictive marker for COVID-19 mortality risk. Nutrients 14, 1407. 35406020 10.3390/nu14071407 PMC9002649

[28] Im JH, Je YS, Baek J, et al. (2020) Nutritional status of patients with COVID-19. Int J Infect Dis 100, 390–393. 32795605 10.1016/j.ijid.2020.08.018 PMC7418699

[29] Skesters A, Kustovs D, Lece A, et al. (2022) Selenium, selenoprotein P, and oxidative stress levels in SARS-CoV-2 patients during illness and recovery. Inflammopharmacology 30, 499–503. 35157169 10.1007/s10787-022-00925-z PMC8853000

[30] Zhou S, Zhang F, Chen F, et al. (2022) Micronutrient level is negatively correlated with the neutrophil-lymphocyte ratio in patients with severe COVID-19. Int J Clin Pract 2022, 6498794. 35685552 10.1155/2022/6498794 PMC9159175

[31] Ozdemir K, Saruhan E, Benli TK, et al. (2022) Comparison of trace element (selenium, iron), electrolyte (calcium, sodium), and physical activity levels in COVID-19 patients before and after the treatment. J Trace Elem Med Biol 73, 127015. 35700624 10.1016/j.jtemb.2022.127015 PMC9150912

[32] Galmés S, Serra F & Palou A (2020) Current state of evidence: influence of nutritional and nutrigenetic factors on immunity in the COVID-19 pandemic framework. Nutrients 12, E2738. 10.3390/nu12092738 PMC7551697 32911778

[33] Notz Q, Herrmann J, Schlesinger T, et al. (2021) Clinical significance of micronutrient supplementation in critically ill COVID-19 patients with severe ARDS. Nutrients 13, 2113. 34203015 10.3390/nu13062113 PMC8235175

[34] Erol SA, Polat N, Akdas S, et al. (2021) Maternal selenium status plays a crucial role on clinical outcomes of pregnant women with COVID-19 infection. J Med Virol 93, 5438–5445. 33951210 10.1002/jmv.27064 PMC8242645

[35] Demircan K, Chillon TS, Sun Q, et al. (2022) Humoral immune response to COVID-19 mRNA vaccination in relation to selenium status. Redox Biol 50, 102242. 35139480 10.1016/j.redox.2022.102242 PMC8810594

[36] Seale LA, Torres DJ, Berry MJ, et al. (2020) A role for selenium-dependent GPX1 in SARS-CoV-2 virulence. Am J Clin Nutr 112, 447–448. 32592394 10.1093/ajcn/nqaa177 PMC7337667

[37] Alexander J, Tinkov A, Strand TA, et al. (2020) Early nutritional interventions with zinc, selenium and vitamin D for raising anti-viral resistance against progressive COVID-19. Nutrients 12, 2358. 32784601 10.3390/nu12082358 PMC7468884

[38] Khatiwada S & Subedi A (2021) A mechanistic link between selenium and coronavirus disease 2019 (COVID-19). Curr Nutr Rep 10, 125–136. 33835432 10.1007/s13668-021-00354-4 PMC8033553

[39] Skalny AV, Rink L, Ajsuvakova OP, et al. (2020) Zinc and respiratory tract infections: perspectives for COVID-19. Int J Mol Med 46, 17–26. 32319538 10.3892/ijmm.2020.4575 PMC7255455

[40] Fakhrolmobasheri M, Mazaheri-Tehrani S, Kieliszek M, et al. (2021) COVID-19 and selenium deficiency: a systematic review. Biol Trace Elem Res 200, 3945–3956. 34739678 10.1007/s12011-021-02997-4 PMC8569840

[41] Zeng HL, Zhang B, Wang X, et al. (2021) Urinary trace elements in association with disease severity and outcome in patients with COVID-19. Environ Res 194, 110670. doi:10.1016/j.envres.2020.110670 33387537 PMC7772999

